# Significant Evolutionary Constraints on Neuron Cells Revealed by Single-Cell Transcriptomics

**DOI:** 10.1093/gbe/evaa054

**Published:** 2020-03-16

**Authors:** Ganlu Hu, Jie Li, Guang-Zhong Wang

**Affiliations:** CAS Key Laboratory of Computational Biology, CAS-MPG Partner Institute for Computational Biology, Shanghai Institute of Nutrition and Health, Shanghai Institutes for Biological Sciences, University of Chinese Academy of Sciences, Chinese Academy of Sciences, Shanghai, China

**Keywords:** evolutionary constraints, single-cell RNA-seq, brain development, cell type, transcriptome evolution

## Abstract

Recent advances in single-cell RNA-sequencing technology have enabled us to characterize a variety of different cell types in each brain region. However, the evolutionary differences among these cell types remain unclear. Here, we analyzed single-cell RNA-seq data of >280,000 cells and developmental transcriptomes of bulk brain tissues. At the single-cell level, we found that the evolutionary constraints on the cell types of different organs significantly overlap with each other and the transcriptome of neuron cells is one of the most restricted evolutionarily. In addition, mature neurons are under more constraints than neuron stem cells as well as nascent neurons and the order of the constraints of various cell types of the brain is largely conserved in different subregions. We also found that although functionally similar brain regions have comparable evolutionary constraints, the early fetal brain is the least constrained and this pattern is conserved in the mouse, macaque, and humans. These results demonstrate the importance of maintaining the plasticity of early brain development during evolution. The delineation of evolutionary differences between brain cell types has great potential for an improved understanding of the pathogenesis of neurological diseases and drug development efforts aimed at the manipulation of molecular activities at the single-cell level.

## Introduction

The brain is the most complex organ known. Its complexity is not only due to the variety of behaviors originating from different neural circuits but also the complex interactions among these circuits. The brain regions involved in complex neural circuits are very distinct at the molecular level (Kang et al. 2011; Hawrylycz et al. 2012, 2015). At the cellular level, the latest single-cell sequencing technology has enabled researchers to reveal the complexity of cell types within a single brain region. Recent studies have found that tens of different cell types can be classified within one brain area, enabling a functional investigation of each cell type (Zeisel et al. 2015, 2018; Tasic et al. 2016).

The functional diversity in different brain regions indicates that the brain endures significant constraints during evolution, because any accumulation of deleterious mutations may cause abnormalities in brain function (Wang et al. 2006; Somel et al. 2013; Brainstorm et al. 2018; Gandal et al. 2018). In fact, the evolutionary rate of brain tissue preferred genes is relatively low (Khaitovich et al. 2005; Wang et al. 2006) and gene expression differences between human and chimpanzee are the smallest in brain compared with other organs (Khaitovich et al. 2005). As biological structure is the basis of their function and brain regions are composed of different cell types, the evolutionary constraints of these cell types might be different from each other as well.

High-expressed genes usually evolve slowly resulting in a negative correlation between expression level and evolutionary rate of a gene at the tissue level (E–R anticorrelation) (Drummond et al. 2006; Liao et al. 2006; Zhang and Yang 2015; Gandal et al. 2018). Several hypotheses have been proposed to explain this phenomenon. Both empirical data and theoretical simulations have provided a large amount of evidence that selection reduces errors in several molecular processes including protein translation, mRNA folding, or protein–protein interactions explain E–R anticorrelation (Zhang and Yang 2015). Therefore, highly expressed genes are more harmful than lowly expressed genes due to these errors (Zhang and Yang 2015). “Evolutionary constraint” refers to the extent that harmful mutations are removed from the population, as it causes molecular errors and reduced fitness. There are considerable variations of E–R anticorrelation among different brain regions and cortical brain regions are subject to more constraints than subcortical brain regions (Tuller et al. 2008). These findings suggest that E–R anticorrelation can be used as a reliable and robust index for assessing the strength of evolutionary constraints at the transcriptome level.

Does E–R anticorrelation exist at the single-cell level? Do different cell types exhibit different E–R anticorrelation levels? Are they under different evolutionary constrains to avoid biological errors? To answer these questions, we first examined the E–R anticorrelation with various single-cell transcriptome data sets and then compared the distribution of this parameter among >700 cell types. Finally, we validated this parameter in ten brain regions as well as during brain development.

## Materials and Methods

### Tissue Level RNA-Seq Data

Tissue level RNA-seq data from adult human brains were downloaded from the Gene Expression Omnibus (GEO) data set with accession number GSE58604 (Wang et al. 2015), previously released by us. Human and macaque brain development and maturation data including 480 and 366 samples, respectively, were downloaded from the PsychENCODE website via the following link: http://development.psychencode.org/ (Li et al. 2018; Zhu et al. 2018). Mouse brain (hypothalamus) development data including 48 samples were obtained from GEO data set GSE21278 (Shimogori et al. 2010). To study the E–R anticorrelation changes during mammalian brain development, we downloaded human–macaque and human–mouse orthologous genes from Ensembl database (through BioMart). We only consider those genes expressed in all three species. This resulted in 10,858 gene sets that were used for further transcriptomic analysis.

### Single-Cell Level RNA-Seq Data Sources

Single-cell RNA-seq data form for mouse primary visual cortex, somatosensory cortex, and hippocampus were downloaded from GEO data set, accession numbers GSE71585 and GSE60361 (Zeisel et al. 2015; Tasic et al. 2016). Single-cell RNA-seq data for human and macaque brain development were downloaded from http://development.psychencode.org/ (Zhu et al. 2018). Single-cell RNA-seq data for mouse cell atlas were obtained from GEO, accession number GSE108097 (Han et al. 2018) and for human LGN and MTG brain cells were downloaded from Allen Brain Atlas via the following link: http://celltypes.brain-map.org/rnaseq. All genes expressed in those data sets were included in the analysis. Single-cell RNA-seq data for dorsal root ganglion (Hu et al. 2016) were downloaded from GEO, accession number GSE71453. The single-cell prefrontal cortex development data were downloaded from GSE104276 (Zhong et al. 2018) and were used to examine the dynamic expression of neurological disease-related genes (supplementary fig. S9, Supplementary Material online).

Detailed information on all the scRNA-seq data analyzed can be found in supplementary table S1, Supplementary Material online.

### Computation of E–R Anticorrelation

We retrieved d*N* and d*S* (*Ka* and *Ks*) of mouse–rat, human–chimpanzee, and human–macaque ortholog genes from European Bioinformatics Institute (https://www.ensembl.org/biomart/martview, last accessed December 10, 2019). Genes with d*S* equal 0 or d*N*/d*S* >2 were not considered for further analysis. For genes with multiple d*N* or d*S* values, averaged d*N* or d*S* values were used. For analyzing evolutionary constraints, genes expressed in at least one cell were retained and cells with <200 expressed genes were removed. The Spearman correlation between expression values and the corresponding d*N* and d*N*/d*S* ratio of each gene were calculated.

### Permutation Test Experiment

For each permutation test, the mean expression levels of each genes for each cell subtypes were resampled. Then, we recalculated their correlation coefficients with d*N* and d*N*/d*S*. About 10,000 experiments were performed in a total of 4,684 cells from both Tasic et al. (2016) and Zeisel et al. (2015) data sets.

### Mouse Cell Atlas Data Analysis and Computation of Corrected E–R Anticorrelation

A total of 242,533 single cells from 38 tissues were included in the initial data set. Then, we removed the cells with <200 expressed genes, leaving 226,456 cells for further analysis. We used the R package “Seurat” for dimension reduction and clustering, following the tutorial at https://satijalab.org/seurat/mca.html. To compare the evolutionary constraints among different tissues and cell types, cells with nonsignificant E–R anticorrelation (adjusted *P *>* *0.05) were removed. To get rid of the effect of detected gene numbers, the residues from the linear regression between E–R anticorrelation and detected gene numbers were used (corrected E–R anticorrelation).

### Gene Dating

The human and mouse gene age data were obtained as reported previously (Zhang et al. 2010). Briefly, the origins of Ensemble v51 protein coding genes were dated by determining the presence and absence of their orthologs along the vertebrate phylogenetic tree. “Young genes” were defined as primate-specific genes (phylogenetic branch ≥8, 1,828 genes) in human- and rodent-specific genes (phylogenetic branch ≥8, 3,111 genes) in mouse, respectively. The remaining genes which originate prior to the primate and rodent split were defined as “old genes.”

### Neurological Disease Genes

We manually obtained 427 Mendelian disease genes from Mendelian Inheritance in Man (OMIM) database (Blekhman et al. 2008). These genes are annotated with 17 different neurological disease phenotypes, including mental retardation, schizophrenia, autism spectrum disease, Alzheimer’s disease, Parkinson’s disease, neurodegeneration, amyotrophic lateral sclerosis, dementia, epilepsy, learning disability, intellectual disability, intellectual development disorder, cognitive impairment, depression, alcohol abuse, sleep disorder, and neurodevelopment disorder. We refer to these genes as neurological disease genes in the main text.

### Statistical Analyses

The Wilcoxon signed ranks test (Wilcoxon test) and Kruskal–Wallis test were used to compare the evolutionary constraints of different tissues/cell types. One-tailed Wilcoxon test was used to calculate the upregulated genes in neuronal cell types. We defined neuronal upregulated genes with fold change >2 and adjusted *P* < 0.05 compared with nonneuronal types. A total of 2,532 and 7,725 neuronal upregulated genes were detected in Tasic et al. (2016) and Zeisel et al. (supplementary table S4, Supplementary Material online). All codes are freely available upon request to the corresponding author.

## Results

### E–R Anticorrelations Widely Exist at the Single-Cell Level

**Fig. 1.**
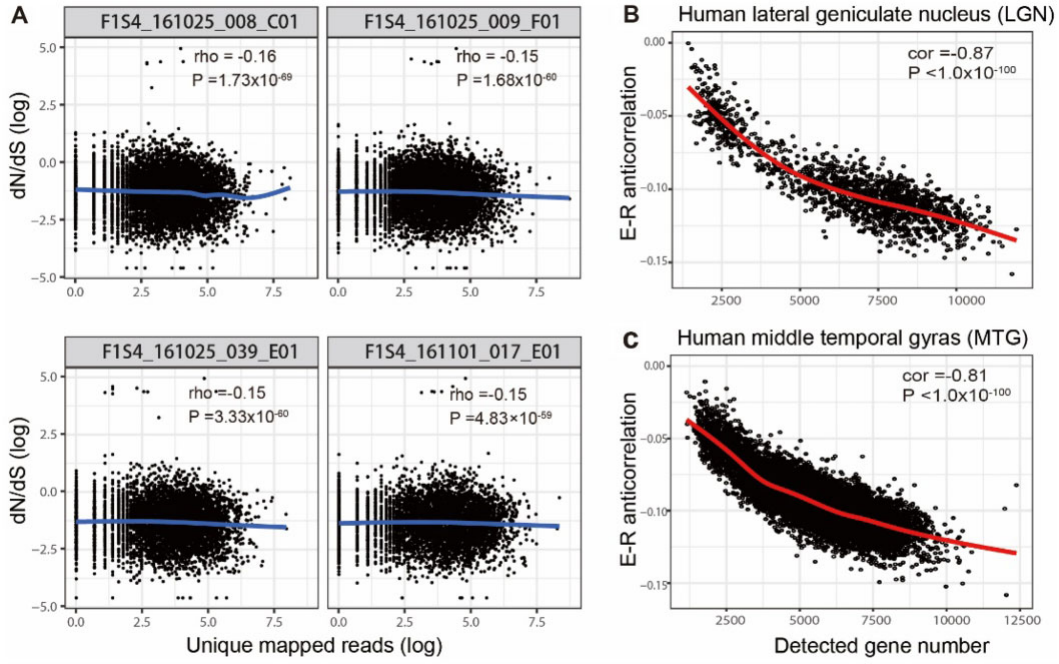
—E–R anticorrelation is prevalent in human brain cells. (*A*) Evolutionary rates (d*N*/d*S*) versus mRNA expression for four representative human LGN cells. mRNA expression level (unique mapped reads) in each single brain cell is presented on the *x* axis (log scaled) and the ratio of d*N* and d*S* (mouse–rat) on the *y* axis (log scaled). rho, Spearman correlation coefficient. (*B* and *C*) E–R anticorrelation decreases as the detected gene numbers increase in both human LGN and MTG brain regions. cor, Pearson correlation coefficient.

Firstly, we examined whether E–R anticorrelation exists at the single-cell level. A total of 44,000 single cells, which were from 4 different types of human and mouse tissues (tumor, blood, brain, and stem cells), were collected (supplementary table S1, Supplementary Material online). Despite the fact that different sequencing platforms, library preparation protocols, and sequencing depths were used, we observed E–R anticorrelation for the majority of cell types (supplementary fig. S1, Supplementary Material online). We also found that the number of genes detected in a sample due to differences in sequencing depth can influence this index (fig. 1*B* and *C*and supplementary fig. S1, Supplementary Material online). When the number of detected genes increases, a stronger E–R anticorrelation is seen for each cell type. Thus, we regressed out the effect of detected gene numbers in the following analysis and refer to this value as E–R anticorrelation (or corrected E–R anticorrelation, see Materials and Methods). Since vertebrate immune cells are under positive selection and accelerating evolutionarily (Hughes and Yeager 1997), we speculate that this might lead to a positive E–R correlation. However, this conjecture could not be confirmed by our results (supplementary fig. S1, Supplementary Material online). Thus, the negative correlation between evolutionary rate and gene expression (E–R anticorrelation) is prevalent at the single-cell level.

### Neuronal Cells Show the Strongest Selective Constraints among Somatic Cells

**Fig. 2.**
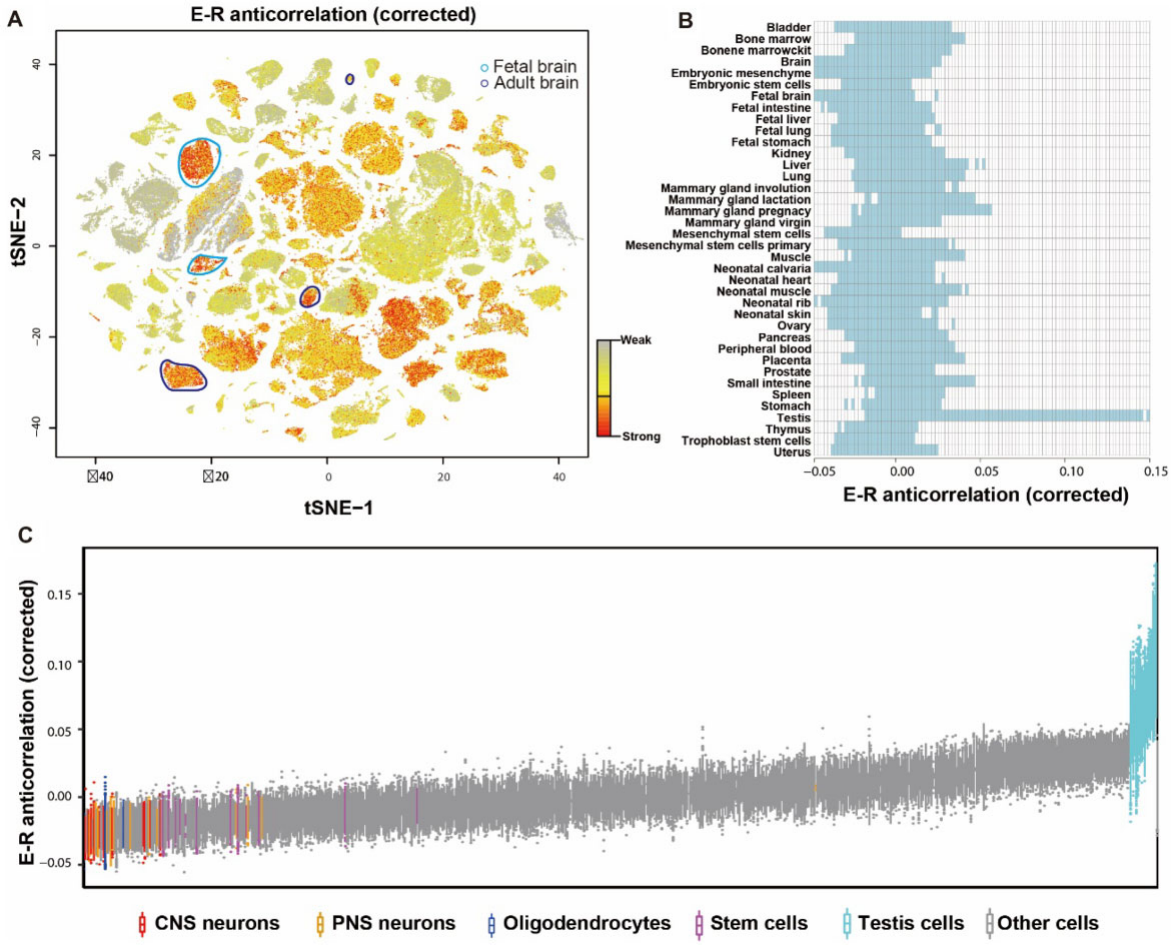
—Evolutionary constraints on transcriptome vary among different tissues and cell types. (*A*) t-SNE was used to perform nonlinear dimensionality reduction on 22K mouse cell atlas transcriptome data, whose position coordinates show the distribution of single cells from 38 tissues. In order to comprehensively study evolutionary constraint levels among single cells from different mouse tissues, E–R anticorrelation was mapped for each cell. Different colors represent the E–R anticorrelation. Orange: low; gray: high. (*B*) The range of corrected E–R anticorrelation among different mouse tissues. (*C*) Boxplot showing the variation of corrected E–R anticorrelation among 760 cell subtypes. Neuronal cells from the central nervous system (CNS) and peripheral nervous system (PNS), oligodendrocyte cells, stem cells, and testis cells are highlighted.

Next, to directly compare the evolutionary constraints of various organs on single-cell level, a recently released mouse single-cell atlas was explored (Han et al. 2018). A total of 226,456 cells from 38 organs were included in the final analysis (supplementary fig. S2*A*, Supplementary Material online). After controlling for detected gene number, we found that the brain transcriptome have stronger E–R anticorrelation than other organs (fig. 2*A* and supplementary fig. S3*A*, Supplementary Material online) (one-sided Wilcoxon test*: P *<* *2.20 × 10^−16^). We then calculated the E–R anticorrelation for each cell type (760 cell types in total) of the 38 organs. We found that significant variations exist in the evolutionary constraints among them, and a great overlap of E–R anticorrelation exists among different cell types of each organ (fig. 2*B* andsupplementary figs. S2*B* and S3*B*, Supplementary Material online). For instance, all organs contain the cell types that fall in the corrected E–R anticorrelation with an interval of −0.055 to 0.173. Even more interesting is the fact that the neuron cell transcriptomes, including neurons from the central nervous system (CNS) and peripheral nervous system (PNS), are under the strongest evolutionary pressure compared with other cell types (fig. 2*C* and supplementary fig. S3*C* and table S2, Supplementary Material online, one-sided Wilcoxon test, *P*_CNS neuron_ <2.20 × 10^−16^, *P*_PNS neuron_ <2.20 × 10^−16^). We also observed a relatively strong E–R anticorrelation in stem cells, indicating that rapidly differentiated cells are sensitive to mutations (fig. 2*C*and supplementary figs. S2*B* and S3*C*, Supplementary Material online, one-sided Wilcoxon test, *P*_stem cells_ < 2.20 × 10^−16^). Therefore, we further analyzed E–R anticorrelation levels in data sets from neuron cells at different stages of maturation (Li et al. 2018). These analyses suggest that mature neurons have a stronger evolutionary constraint than nascent neurons and neuron stem cells while rapidly evolving genes tend to be expressed in the earlier stages of maturation (supplementary fig. S4*C*, Supplementary Material online). These results emphasize the importance of investigating the evolutionary differences of organs based on their cell types.

### The Strength of Evolutionary Constraints in Different Cell Types Is Conserved

**Fig. 3.**
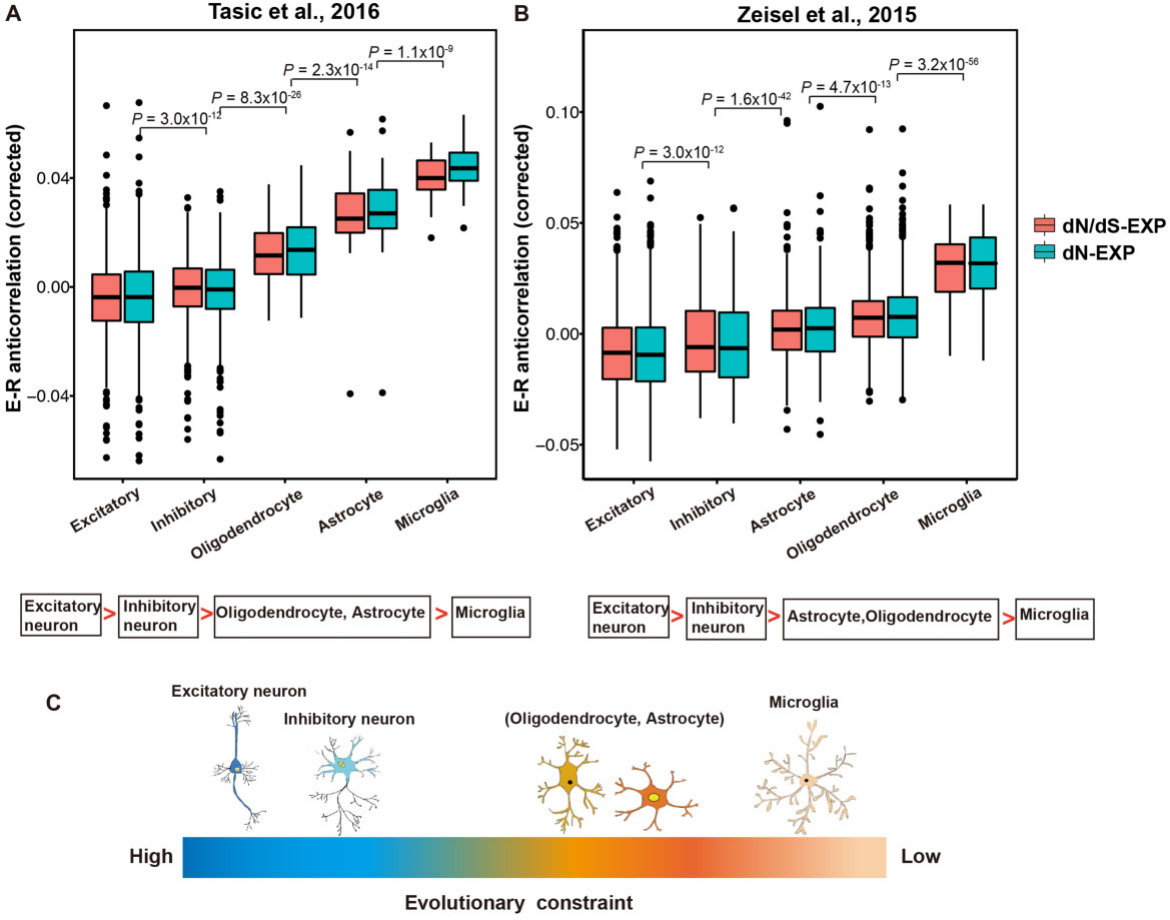
—Different evolutionary constraints in distinct brain cell types. (*A* and *B*) The comparison of E–R anticorrelations among different brain cell types for Tasic et al. (2016) and Zeisel et al. data sets. *P* values from pairwise comparisons are labeled. (*C*) Schematic diagram showing the conservative order of different evolutionary constraints in distinct cell types in the two brain regions.

To better distinguish the evolutionary constraints of different cell types within neuronal tissue, two data sets with high-sequencing quality were employed (Zeisel et al. 2015; Tasic et al. 2016). In both data sets, significant differences of E–R anticorrelation among cell types were observed (fig. 3*A* and *B*). Interestingly, excitatory and inhibitory neurons show stronger E–R anticorrelation than nonneuronal cells, even after controlling for the detected gene number (fig. 3*A* and *B* andsupplementary table S3, Supplementary Material online). E–R anticorrelation is stronger in excitatory neurons than in inhibitory neurons, implicating stronger evolutionary constraints on the transcriptome of excitatory neurons. We further noticed that the strength of evolutionary constraints rank for different cell types by and large is conserved between these two data sets (excitatory neuron > inhibitory neuron > oligodendrocyte and astrocyte > microglia) implying that the selective pressure acting on different cell types is relatively stable in different brain regions (fig. 3*C*, one-tailed Wilcoxon test, *P *<* *0.0001 in all the comparison). This ranking order was further validated by the RNA-sequencing transcriptome data which were from fluorescence-activated cell sorting purified brain cells with higher read depth (Zhang et al. 2014) (supplementary fig. S5, Supplementary Material online), implying the robustness of our analysis. As both excitatory and inhibitory neurons can be classified into tens of subtypes, more investigations are required to further clarify whether the transcriptome of certain subclasses of inhibitory neurons is more constrained than that of excitatory neurons at some specific developmental periods or in some specific brain regions. Further, we found that the correlation between E–R anticorrelation and mean expression level in Tasic et al. (2016) data set is not significant (r = 0.054, *P* = 0.82, supplementary fig. S6*A*, Supplementary Material online). And, in the data set of Zeisel et al. (2015), after controlling for the effect of detected gene number, there is no significant correlation between mean expression and E–R anticorrelation as well (*r* = −0.0079, *P* = 0.66, supplementary fig. S6*B*, Supplementary Material online). Therefore, our results cannot be explained by the difference of mean expression levels in different cell types.

We next checked the E–R anticorrelation patterns among different sensory neuronal types of dorsal root ganglion and observed significantly weaker constraints in nociceptors neurons compared with other two neuronal cell types (supplementary fig. S7, Supplementary Material online). We conclude that the evolutionary constraints are diverse among different cell types in both central nervous cells and peripheral nervous cells.

### Evolutionary Constraints of Single Cells Are Stronger Than Expected

Are the evolutionary constraints of each cell type in the nervous tissue stronger than expected by chance? To answer this question, 10,000 randomization experiments were preformed, where the expression of each gene was shuffled and their E–R anticorrelations were recalculated. We found that the E–R anticorrelation of each cell type is much stronger than in the permutation experiments (supplementary fig. S8, Supplementary Material online, one-tailed Wilcoxon test, *P *<* *0.05 for all the 12 comparisons).

### Both Neuronal Upregulated Genes and Neurological Disease-Related Genes Contribute to the Strong Evolutionary Constraints of Neurons

We next estimated the effect of neuronal upregulated genes on the high evolutionary constraints of neurons from Tasic et al. (2016) data set and found that the evolutionary rate of those genes (d*N* and d*N*/d*S*) is lower than other expressed genes in neurons (for d*N*, *P *=* *7.25 × 10^−17^; for d*N*/d*S*, *P *=* *9.20 × 10^−15^, one-tailed Wilcoxon test). After removing those genes, the E–R anticorrelation of neuronal cells is decreased (supplementary table S4, Supplementary Material online, for d*N*-Exp, *P *=* *3.67 × 10^−27^; for d*N*/d*S*-Exp, *P *=* *1.26 × 10^−39^, one-tailed Wilcoxon test). This effect also exists for neurological disease genes (for d*N*-Exp, *P *=* *7.78 × 10^−6^; for d*N*/d*S*-Exp, *P *=* *3.92 × 10^−7^, one-tailed Wilcoxon test) and similar results were found using the data set from Zeisel et al. (2015). This indicates that both neuronal upregulated genes and neurological disease genes are under higher selective pressure and contribute to the greater constraints of neuronal cells in evolution.

We also found that there are more genes expressed in adult brain (525.34 ± 258.30) than fetal brain (453.00 ± 147.10) (*P *=* *2.40 × 10^−9^, one-tailed Wilcoxon test) and neurological disease genes have higher expression levels in the adult brain than in the fetal brain (*P *=* *2.25 × 10^−25^, one-tailed Wilcoxon test) (supplementary fig. S9*A*, Supplementary Material online). Thus, the adult brain is generally more susceptible to neurological diseases. Finally, the expression of these pathogenic genes is slightly increased during the development of prefrontal cortex (supplementary fig. S9*B*, Supplementary Material online).

### Early Brain Development Exhibits the Least Evolutionary Constraints

**Fig. 4.**
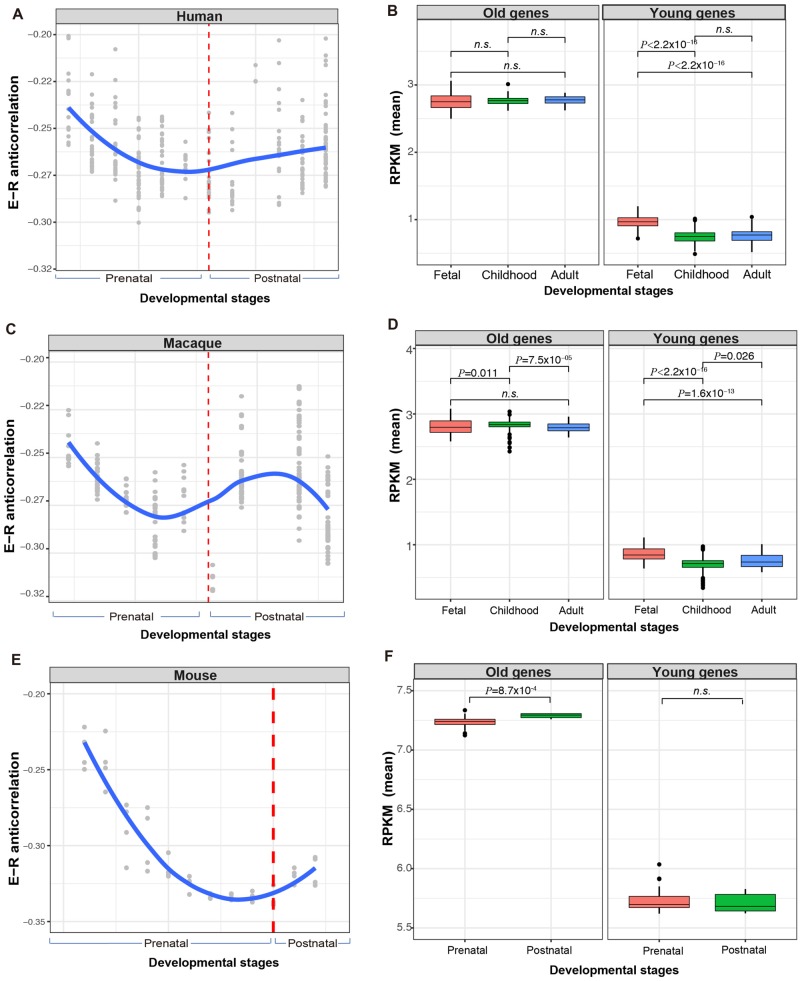
—Early fetal brain is the least constrained during development. (*A*, *C*, and *E*) Dynamic changes of evolutionary constraints in human, macaque, and mouse cortex during development. Red dash line represents “birth.” (*B*, *D*, and *F*) Expression levels of “young genes” and “old genes” in mammalian brains at different developmental periods. *P* values from pairwise comparisons are indicated.

In order to further study the evolutionary constraint of brain neurons on the tissue level and temporal scale, we next calculated E–R anticorrelation in the previously reported ten language-related Brodmann areas (Wang et al. 2015), which are all from healthy adult samples. As shown in supplementary fig. S10, Supplementary Material online, there are no significant differences in E–R anticorrelations among these ten brain regions (Kruskal–Wallis χ^2^ = 3.69, *P *=* *0.93), indicating that evolutionary constraints are very similar in functionally similar brain regions. This result is not in contradiction with a previous report as more distinct brain regions were used before (Tuller et al. 2008). We then studied how evolutionary constraints change during brain development by analyzing the recently published PsychENCODE data, which profiled the transcriptome of distinct human brain regions from different developmental stages (Li et al. 2018; Zhu et al. 2018). Our results suggest that the evolutionary constraints are the weakest in the early infancy brain and then increase during development (fig. 4*A*). This pattern is consistent for different subregions of the human brain (supplementary fig. S11, Supplementary Material online) and is conserved in both monkey and mouse (fig. 4*C* and *E*), which indicates that the early development of the mammalian brain requires more plasticity and thus fewer constraints during evolution. Additionally, consistent with a previous report (Zhang et al. 2011), we found that the young genes are upregulated in the fetal brain of humans and monkeys, but not in those of the mouse (fig. 4*B*, *D*, and *F*).

## Discussion

By using single-cell sequencing data from >280,000 cells, we showed that E–R anticorrelation is well established for all cell types. At the cellular level, the selection constraints in different cell types vary with differentiated cells being under more constraints. Thus, there is no direct relationship between the selective constraints and the physical distance of the cells and adjacent cell types may have great variations in terms of their evolutionary constraints. We further found that the evolutionary constraints of neuronal cells are nearly always the strongest among different somatic cell types. At last, we analyzed the evolutionary constraints of brain at different developmental stages on tissue level. Although functionally similar brain regions have similar constraints, the early fetal brain exhibited the weakest evolutionary constraints and this pattern is conserved across three species.

The development of single-cell RNA-sequencing technology has allowed us to isolate and compare neuronal cells between species on a large scale. A recent comparison of different cell types between the cortex of two reptilian species and those of the mouse/human has suggested that novel excitatory neurons are generated while inhibitory neurons are mostly conserved during the evolution of amniotes (Tosches et al. 2018), which has highlighted different evolutionary pathways of excitatory and inhibitory neurons. In this study, our results demonstrate that neuronal cells have stronger evolutionary constraints than nonneuronal cells, implicating that more functional divergence of neuronal cells exists compared with nonneuronal cells in the nervous systems. The stronger constraints in neurons are partly due to upregulated neuronal genes and neurological disease-related genes that evolve more slowly than other genes in the cell. Our results are consistent with the recent finding that oligodendrocytes have undergone an accelerated evolution compared with neurons in the human lineage (Berto et al. 2019). Interestingly, the study by Berto et al. (2019) observed that human-specific genes in oligodendrocytes tend to be related to neuropsychiatric disorders, highlighting the importance of myelination and oligodendrocytes for the pathobiology of these disorders (Berto et al. 2019).

We found that early mammalian brain evolution was highly plastic compared with later life span stages. Consistent with an earlier study (Zhang et al. 2011), the human and monkey brains have more “young” genes upregulated than the mouse brain, demonstrating accelerated evolution of the primate infant brain. Additionally, a recent study has shown that the most considerable transcriptomic divergences between the human and macaque brains occur during the early stages of development (Zhu et al. 2018), which is consistent with our results. In summary, we have shown that the transcriptional plasticity of the early brain may be one of the key factors determining the direction of mammalian brain evolution.

Finally, more detailed evolutionary differences between cell types of different organs are revealed with single-cell sequencing technology. Since even within the same organ various selective constraints among different cell types exist and those constraints can overlap, it may be of particular importance to classify and investigate the transcriptional differences of distinct cell types across multiple organs rather than within a specific organ. Thus, understanding the evolutionary differences between cell types has great potential for shedding light on the pathogenesis of neurological diseases and contributing to the development of drugs based on the molecular activities at the single-cell level. 

## Supplementary Material

evaa054_Supplementary_Data

## References

[evaa054-B1] Berto S, et al 2019 Accelerated evolution of oligodendrocytes in the human brain. Proc Natl Acad Sci USA. 116(48):24334–24342. 31712436 10.1073/pnas.1907982116 PMC6883816

[evaa054-B2] Blekhman R, et al 2008 Natural selection on genes that underlie human disease susceptibility. Curr Biol. 18(12):883–889. 18571414 10.1016/j.cub.2008.04.074 PMC2474766

[evaa054-B3] Brainstorm C, et al 2018 Analysis of shared heritability in common disorders of the brain. Science 360(6395):eaap8757. 10.1126/science.aap8757 PMC6097237 29930110

[evaa054-B4] Drummond DA, Raval A, Wilke CO. 2006 A single determinant dominates the rate of yeast protein evolution. Mol Biol Evol. 23(2):327–337. 16237209 10.1093/molbev/msj038

[evaa054-B5] Gandal MJ, et al 2018 Shared molecular neuropathology across major psychiatric disorders parallels polygenic overlap. Science 359(6376):693–697. 29439242 10.1126/science.aad6469 PMC5898828

[evaa054-B6] Han X, et al 2018 Mapping the mouse cell atlas by Microwell-seq. Cell 172(5):1091–1107 e1017. 29474909 10.1016/j.cell.2018.02.001

[evaa054-B7] Hawrylycz M, et al 2015 Canonical genetic signatures of the adult human brain. Nat Neurosci. 18(12):1832–1844. 26571460 10.1038/nn.4171 PMC4700510

[evaa054-B8] Hawrylycz MJ, et al 2012 An anatomically comprehensive atlas of the adult human brain transcriptome. Nature 489(7416):391–399. 22996553 10.1038/nature11405 PMC4243026

[evaa054-B9] Hu G, et al 2016 Single-cell RNA-seq reveals distinct injury responses in different types of DRG sensory neurons. Sci Rep. 6(1):31851. 27558660 10.1038/srep31851 PMC4997251

[evaa054-B10] Hughes AL, Yeager M. 1997 Molecular evolution of the vertebrate immune system. Bioessays 19(9):777–786. 9297968 10.1002/bies.950190907

[evaa054-B11] Kang HJ, et al 2011 Spatio-temporal transcriptome of the human brain. Nature 478(7370):483–489. 22031440 10.1038/nature10523 PMC3566780

[evaa054-B12] Khaitovich P, et al 2005 Parallel patterns of evolution in the genomes and transcriptomes of humans and chimpanzees. Science 309(5742):1850–1854. 16141373 10.1126/science.1108296

[evaa054-B13] Li M, et al 2018 Integrative functional genomic analysis of human brain development and neuropsychiatric risks. Science 362(6420):eaat7615. 30545854 10.1126/science.aat7615 PMC6413317

[evaa054-B14] Liao BY, Scott NM, Zhang J. 2006 Impacts of gene essentiality, expression pattern, and gene compactness on the evolutionary rate of mammalian proteins. Mol Biol Evol. 23(11):2072–2080. 16887903 10.1093/molbev/msl076

[evaa054-B15] Shimogori T, et al 2010 A genomic atlas of mouse hypothalamic development. Nat Neurosci. 13(6):767–775. 20436479 10.1038/nn.2545 PMC4067769

[evaa054-B16] Somel M, Liu X, Khaitovich P. 2013 Human brain evolution: transcripts, metabolites and their regulators. Nat Rev Neurosci. 14(2):112–127. 23324662 10.1038/nrn3372

[evaa054-B17] Tasic B, et al 2016 Adult mouse cortical cell taxonomy revealed by single cell transcriptomics. Nat Neurosci. 19(2):335–346. 26727548 10.1038/nn.4216 PMC4985242

[evaa054-B18] Tosches MA, et al 2018 Evolution of pallium, hippocampus, and cortical cell types revealed by single-cell transcriptomics in reptiles. Science 360(6391):881–888. 29724907 10.1126/science.aar4237

[evaa054-B19] Tuller T, Kupiec M, Ruppin E. 2008 Evolutionary rate and gene expression across different brain regions. Genome Biol. 9(9):R142. 18811952 10.1186/gb-2008-9-9-r142 PMC2592720

[evaa054-B20] Wang GZ, et al 2015 Correspondence between resting-state activity and brain gene expression. Neuron 88(4):659–666. 26590343 10.1016/j.neuron.2015.10.022 PMC4694561

[evaa054-B21] Wang HY, et al 2006 Rate of evolution in brain-expressed genes in humans and other primates. PLoS Biol. 5(2):e13. 10.1371/journal.pbio.0050013 PMC1717015 17194215

[evaa054-B22] Zeisel A, et al 2015 Brain structure. Cell types in the mouse cortex and hippocampus revealed by single-cell RNA-seq. Science 347(6226):1138–1142. 25700174 10.1126/science.aaa1934

[evaa054-B23] Zeisel A, et al 2018 Molecular architecture of the mouse nervous system. Cell 174(4):999–1014 e1022. 30096314 10.1016/j.cell.2018.06.021 PMC6086934

[evaa054-B24] Zhang J, Yang JR. 2015 Determinants of the rate of protein sequence evolution. Nat Rev Genet. 16(7):409–420. 26055156 10.1038/nrg3950 PMC4523088

[evaa054-B25] Zhang Y, et al 2014 An RNA-sequencing transcriptome and splicing database of glia, neurons, and vascular cells of the cerebral cortex. J Neurosci. 34(36):11929–11947. 25186741 10.1523/JNEUROSCI.1860-14.2014 PMC4152602

[evaa054-B26] Zhang YE, Landback P, Vibranovski MD, Long M. 2011 Accelerated recruitment of new brain development genes into the human genome. PLoS Biol. 9(10):e1001179. 22028629 10.1371/journal.pbio.1001179 PMC3196496

[evaa054-B27] Zhang YE, Vibranovski MD, Landback P, Marais GA, Long M. 2010 Chromosomal redistribution of male-biased genes in mammalian evolution with two bursts of gene gain on the X chromosome. PLoS Biol. 8(10):e1000494. 20957185 10.1371/journal.pbio.1000494 PMC2950125

[evaa054-B28] Zhong S, et al 2018 A single-cell RNA-seq survey of the developmental landscape of the human prefrontal cortex. Nature 555(7697):524–528. 29539641 10.1038/nature25980

[evaa054-B29] Zhu Y, et al 2018 Spatiotemporal transcriptomic divergence across human and macaque brain development. Science 362(6420):eaat8077. 30545855 10.1126/science.aat8077 PMC6900982

