# Supplementary material for: Significant Evolutionary Constraints on Neuron Cells Revealed by Single-Cell Transcriptomics
Source: Genome Biol Evol. 2020 Mar 16;12(4):300–8. doi: 10.1093/gbe/evaa054 (PMC7186789; doi:10.1093/gbe/evaa054)
Supplement: evaa054_Supplementary_Data [file evaa054_supplementary_data.zip › Supplementary Materials 12-13-v2.docx]

**Supplementary Material**

**Table S1.** Single-cell RNA-seq datasets analyzed in this study.

**Table S2.** E-R anticorrelation of 760 mouse cell types across different organs.

**Table S3.** Comparison of E-R anticorrelation values among different cell types in the brain.

**Table S4.** List of neuronal cell upregulated genes in both Tasic et al. 2016 and Zeisel et al. 2015.

**Supplementary codes.** R codes used in the key analysis of the manuscript, including the calculation of E-R anticorrelation

**Supplementary Figures**


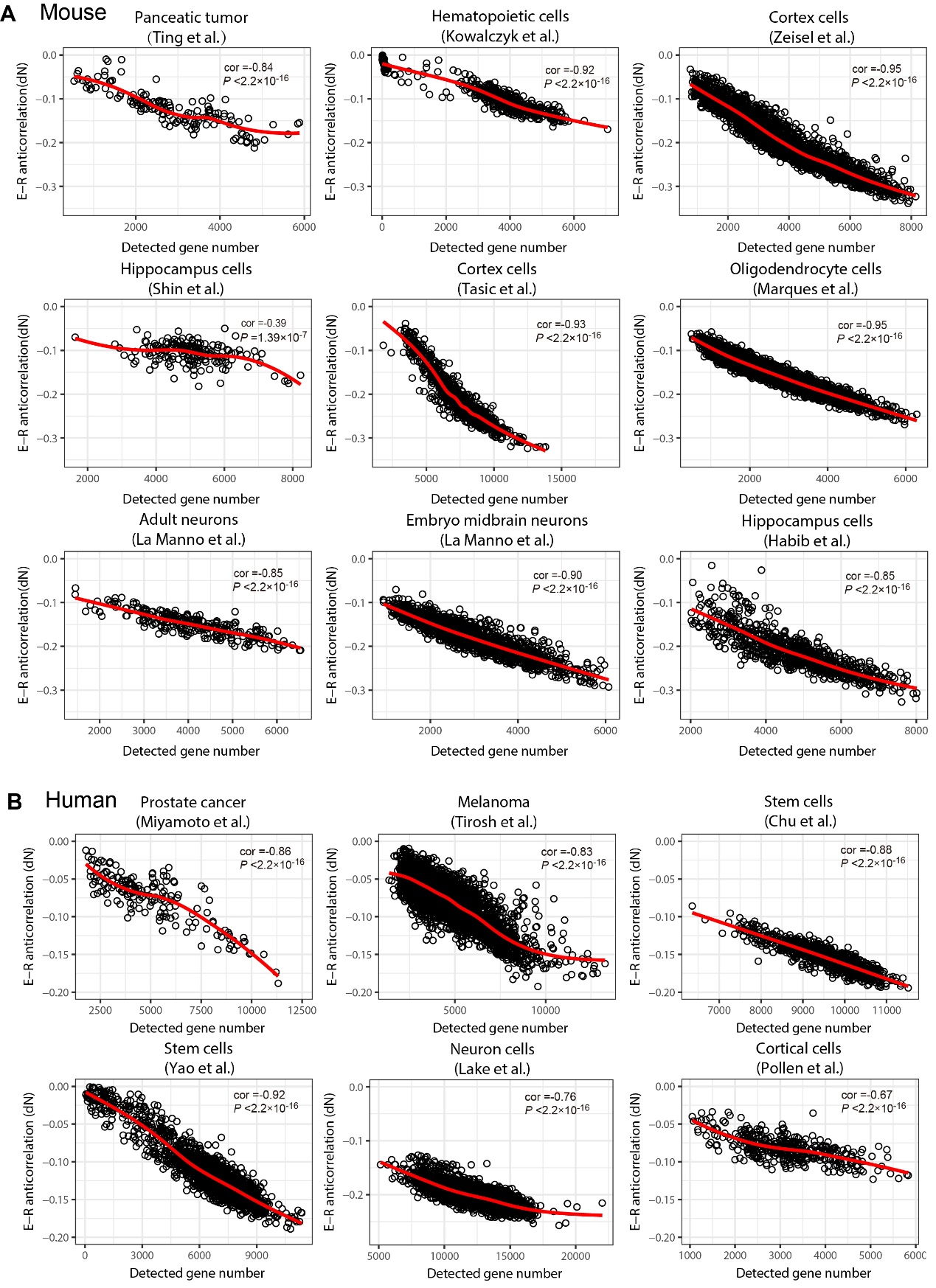


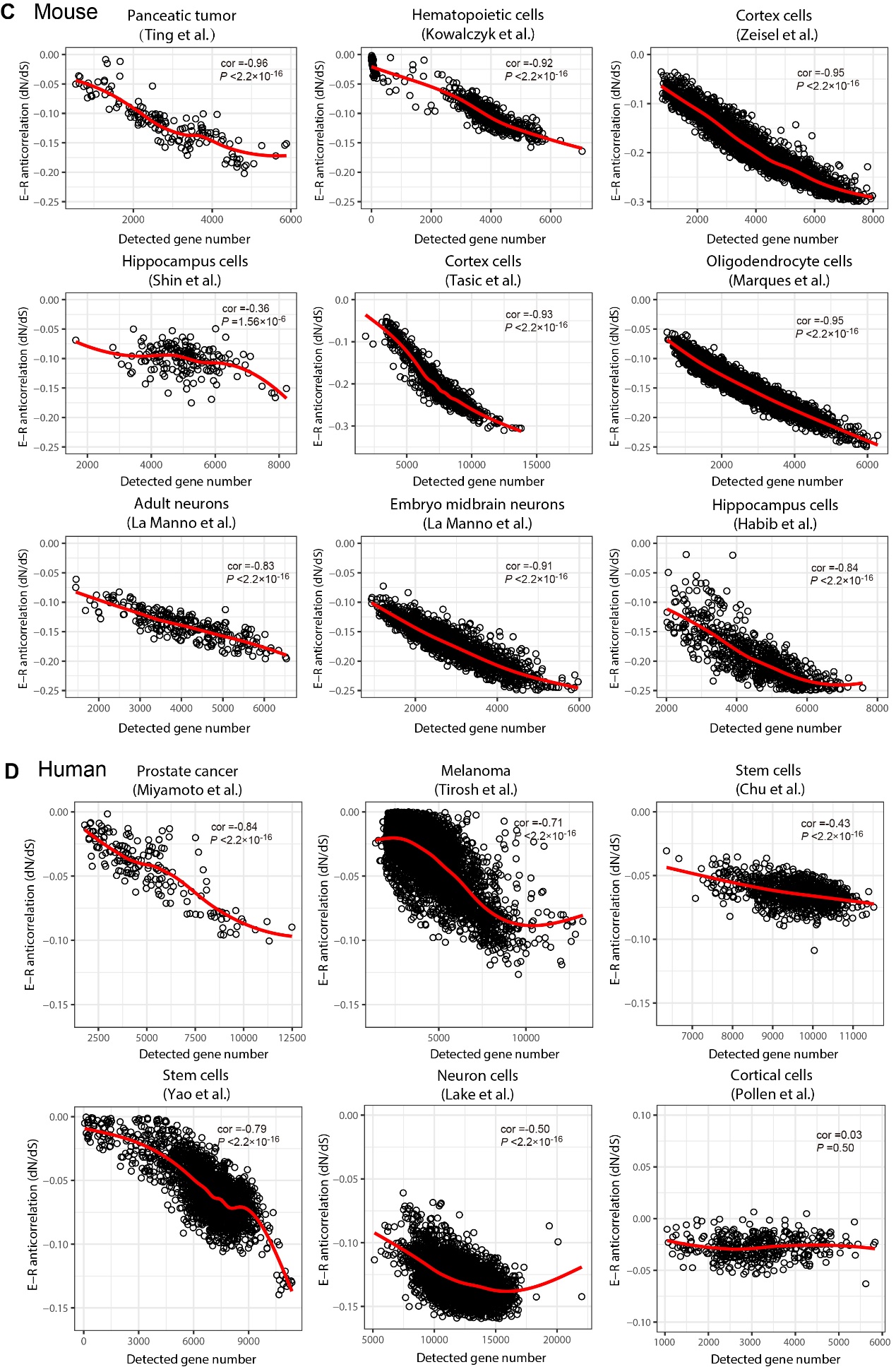


Fig S1. E-R anticorrelations pervasively exist at single cell level. Relationship of number of genes detected in different cell types and their coordinated E-R anticorrelation for both mouse (A,C) and human (B,D) single cells.


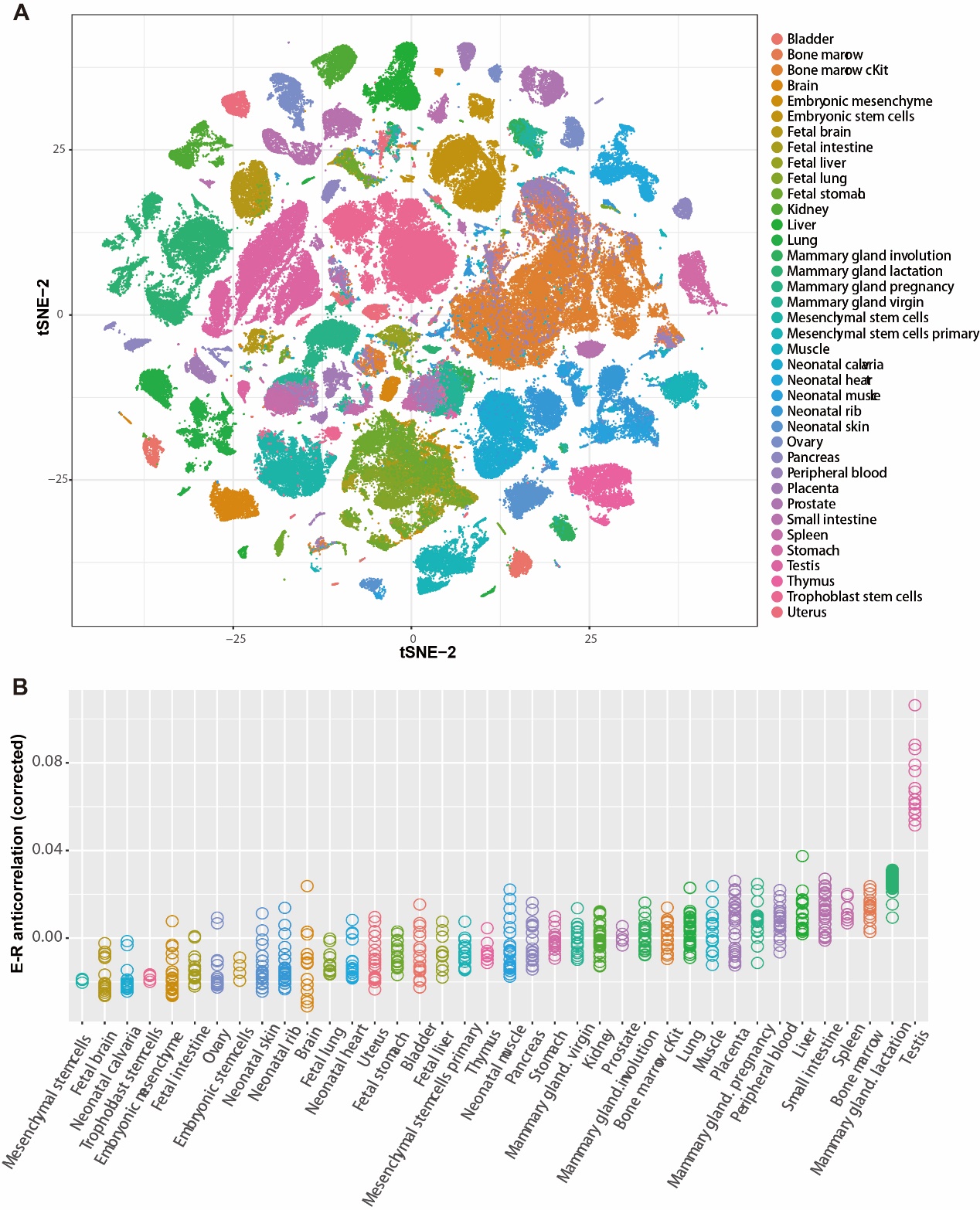


Fig S2. E-R anticorrelations vary among different mouse tissues. (A) t-SNE shows the separation of 38 different mouse tissues using scRNA-seq data from Han et al. (B) Dotplot shows the variation of corrected E-R anticorrelation level among different mouse tissues as well as different cell types. Each dot represents a cell subtype.


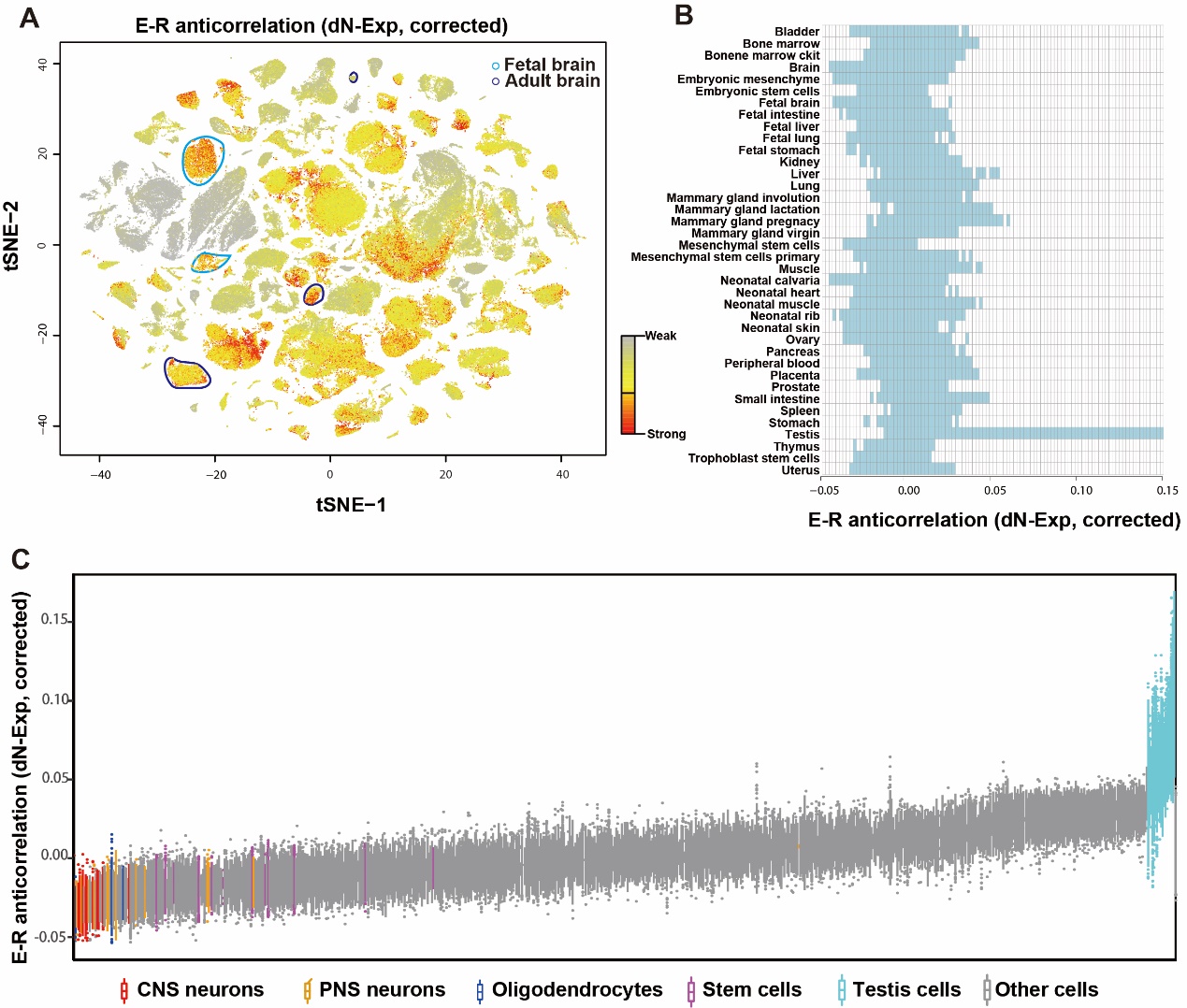


Fig S3. E-R anticorrelations vary among different tissues and cell types. (A) t-SNE shows the distribution of 38 tissues of the mouse cell atlas data. E-R anticorrelation (dN-Exp, corrected) among different mouse tissues were mapped to each cell. Different colors represent the normalized values. Orange: the lowest value; gray: the highest value. (B) The range of E-R anticorrelation (dN-Exp, corrected) among different mouse tissues. (C) Boxplot shows the variation of E-R anticorrelation (dN-Exp, corrected) among 760 cell subtypes. Neuronal cells from the central nervous system and peripheral nervous system, oligodendrocyte cells, stem cells and testis cells are highlighted.


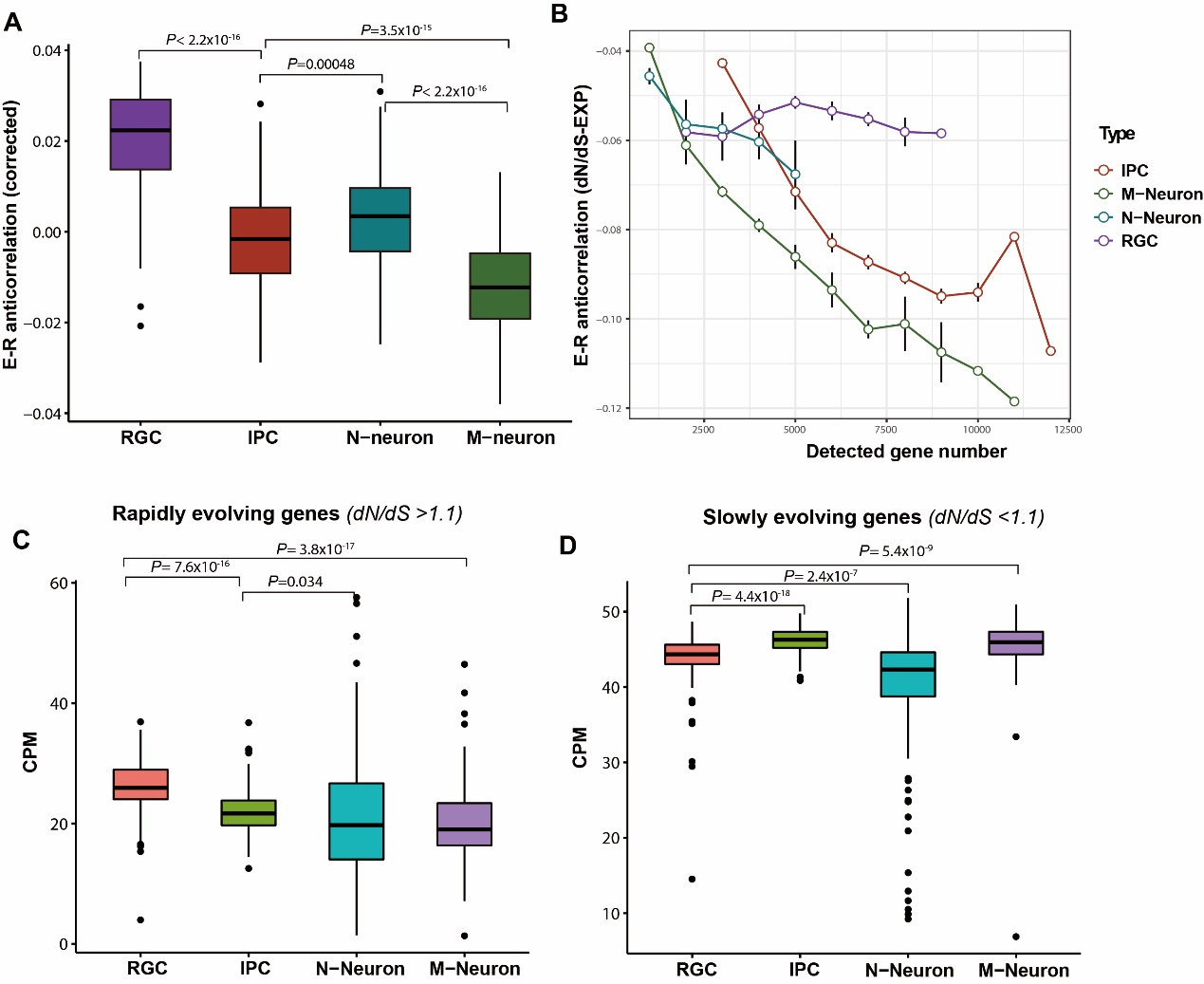


Fig S4. Neuronal stem cells and nascent neurons are under lower evolutionary constraints than mature neurons. (A) Comparison of E-R anti-correlations among neuronal cells at different differentiation stages. (B) Relationship between number of detected genes and E-R anticorrelations. (C) The expression levels of rapidly evolving genes among neuronal cells at different developmental stages. (D) The expression levels of slowly evolving genes among neuronal cells at different developmental stages. Rapidly evolving genes, genes with dN/dS value >1.1: Slowly evolving genes, genes with dN/dS value <1.1. RGC, radial glia cells: IPC, intermediate progenitor cells: N-neuron, nascent neuron: M-neuron, mature neuron.


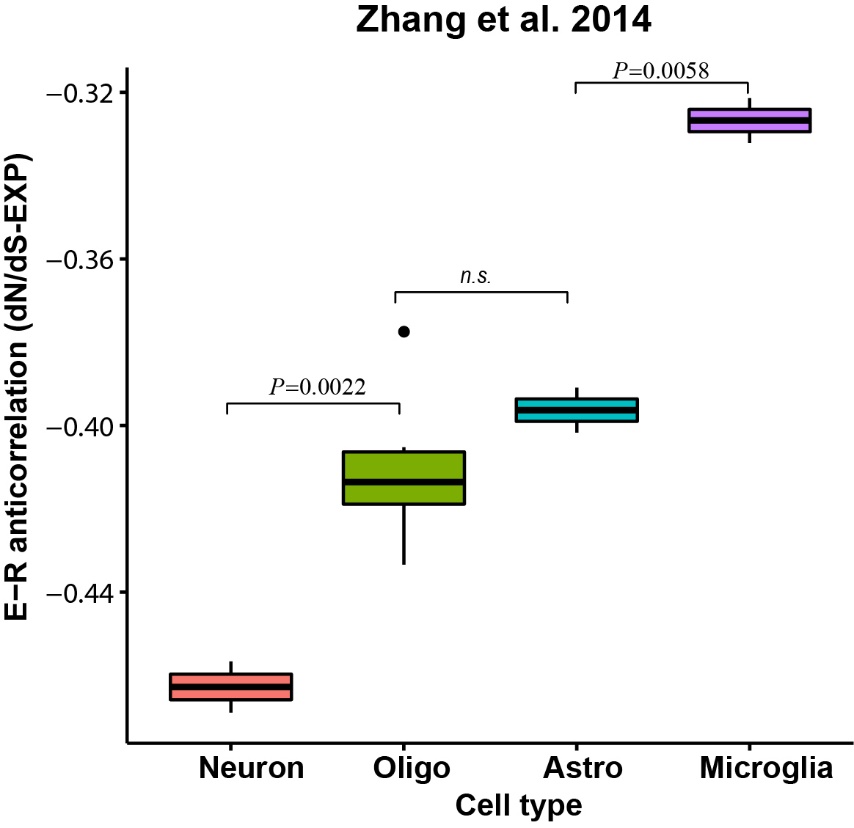


Fig S5. Differential E-R anticorrelations among distinct brain cell types on bulk cells by using Zhang et al. data (Zhang, et al. 2014). *P* values from pairwise t-test are indicated.


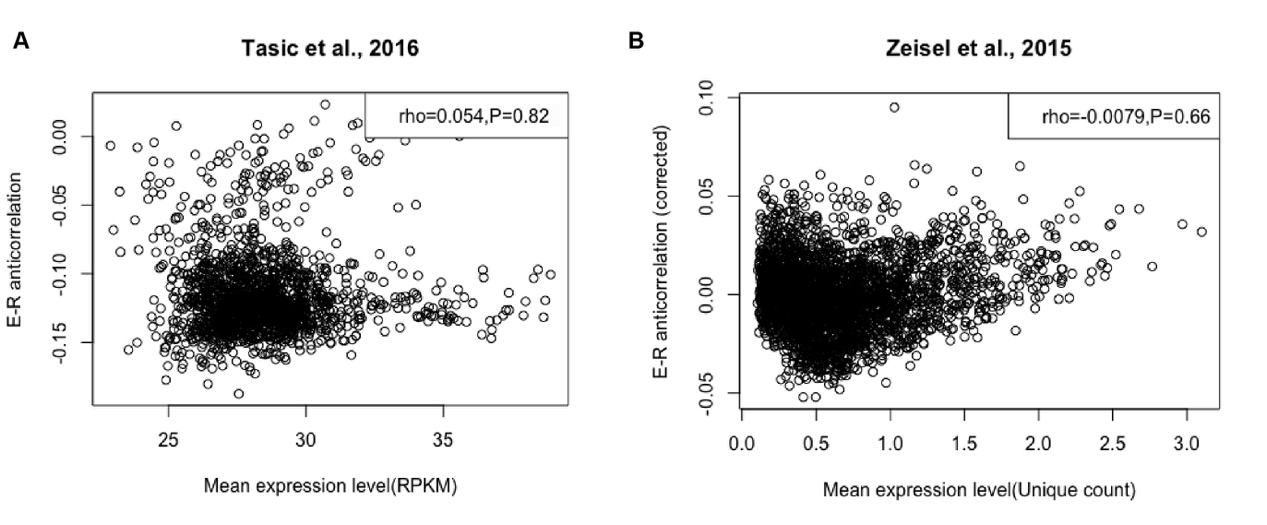


Fig S6. Relationship of mean expression level versus E-R anticorrelation on single cell level for Tasic et al.,2016 (A) and Zeisel et al.,2015 (B) dataset.


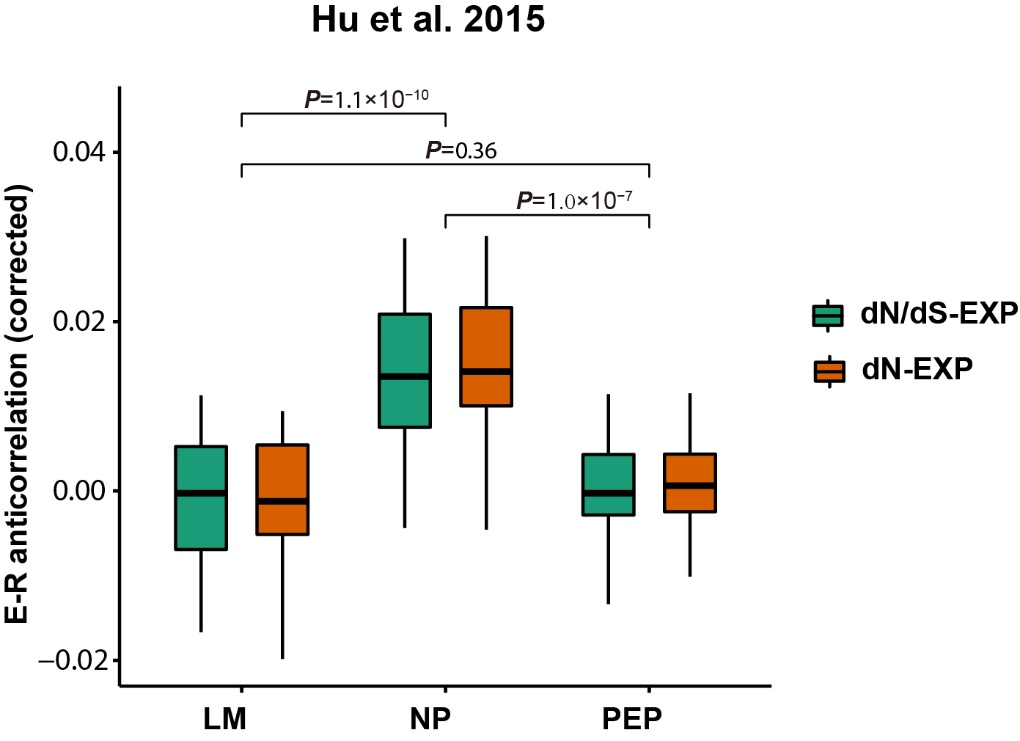


Fig S7. Differential E-R anticorrelations among peripheral neuronal cells. Boxplot shows corrected E-R anticorrelation levels among different sensory neuron subtypes. *P* values of corrected E-R anticorrelation levels from multiple comparisons are indicated. LM, large myelinated neuron. NP, nociceptors. PEP, peptidergic nociceptors.


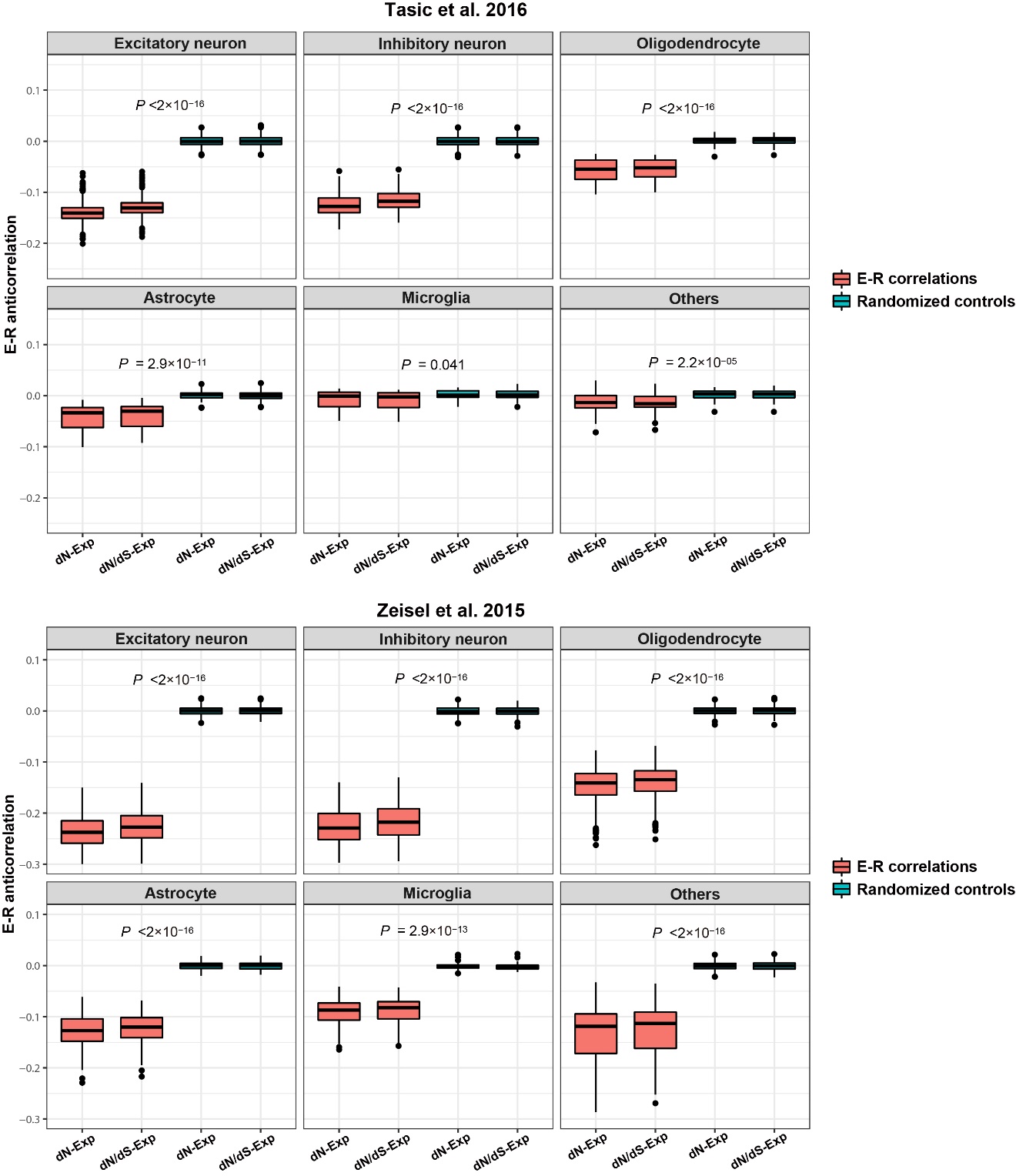


Fig S8. E-R anticorrelation levels among different brain cell types are stronger than random controls. Boxplots show the comparison of E-R anticorrelation levels among different brain cell types in Tasic et al. (A) and Zeisel et al. (B) datasets. E-R correlations: correlations between gene expression levels and evolutionary rates (both dN and dN/dS), Randomized control: correlations between 10,000 times shuffled genes expression levels and dN or dN/dS. *P* values of Wilcoxon rank sum test are indicated.


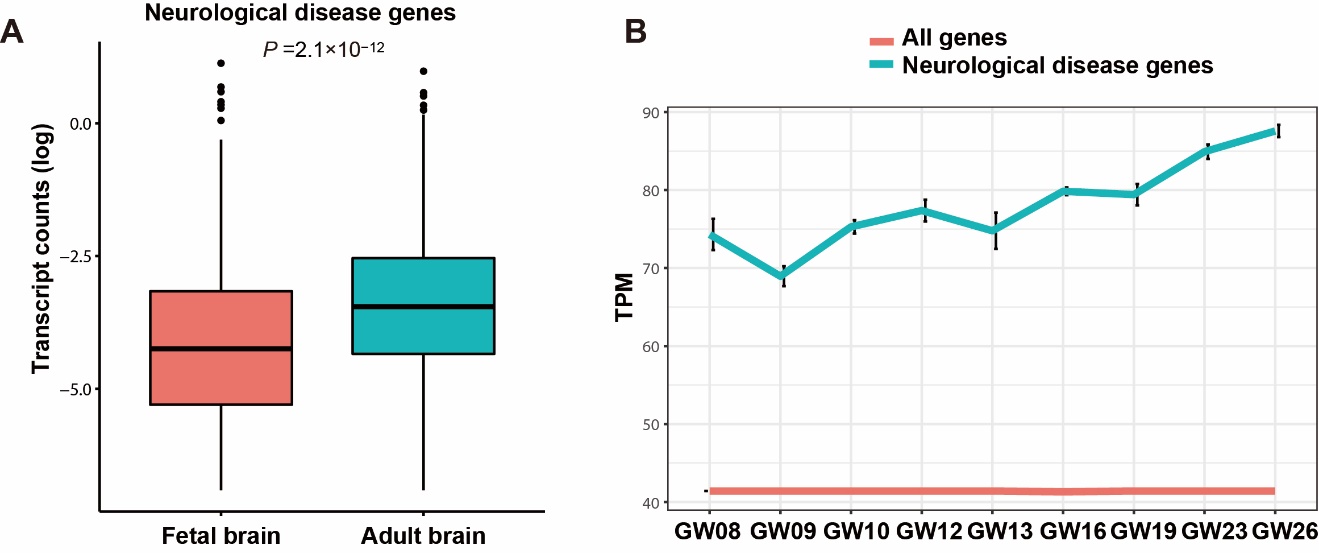


Fig S9. Neurological disease genes have increased expression during brain development. (A) Boxplots show significantly higher levels of expression of neurological disease genes in adult brain than in fetal brain, *P* <2.1×10^-12^, Wilcoxon rank sum test. (B) The mean expression of neurological diseases is increased during the early development stage of the human frontal cortex. TPM, transcripts per million reads. GW, gestational weeks.


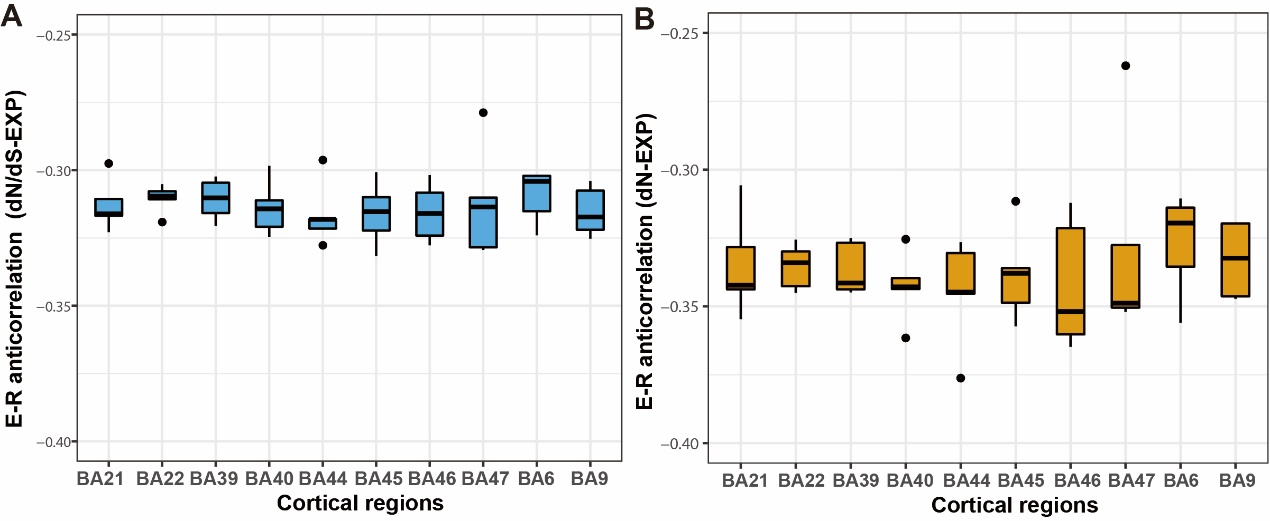


Fig S10. Similar level of E-R anticorrelations among different adult brain regions. (A) Correlation between evolutionary rates (dN/dS-Exp) versus the expression levels in 10 cortical brain regions. The correlation coefficients are not significantly different among different Brodmann regions (Kruskal-Wallis chi-squared = 4.7097, df = 9, *P* =0.86). (B) Correlation between evolutionary rates (dN-Exp) versus the expression levels in 10 cortical brain regions. The correlation coefficients are not significantly different among different Brodmann regions (*P* > 0.15, one-way ANOVA).


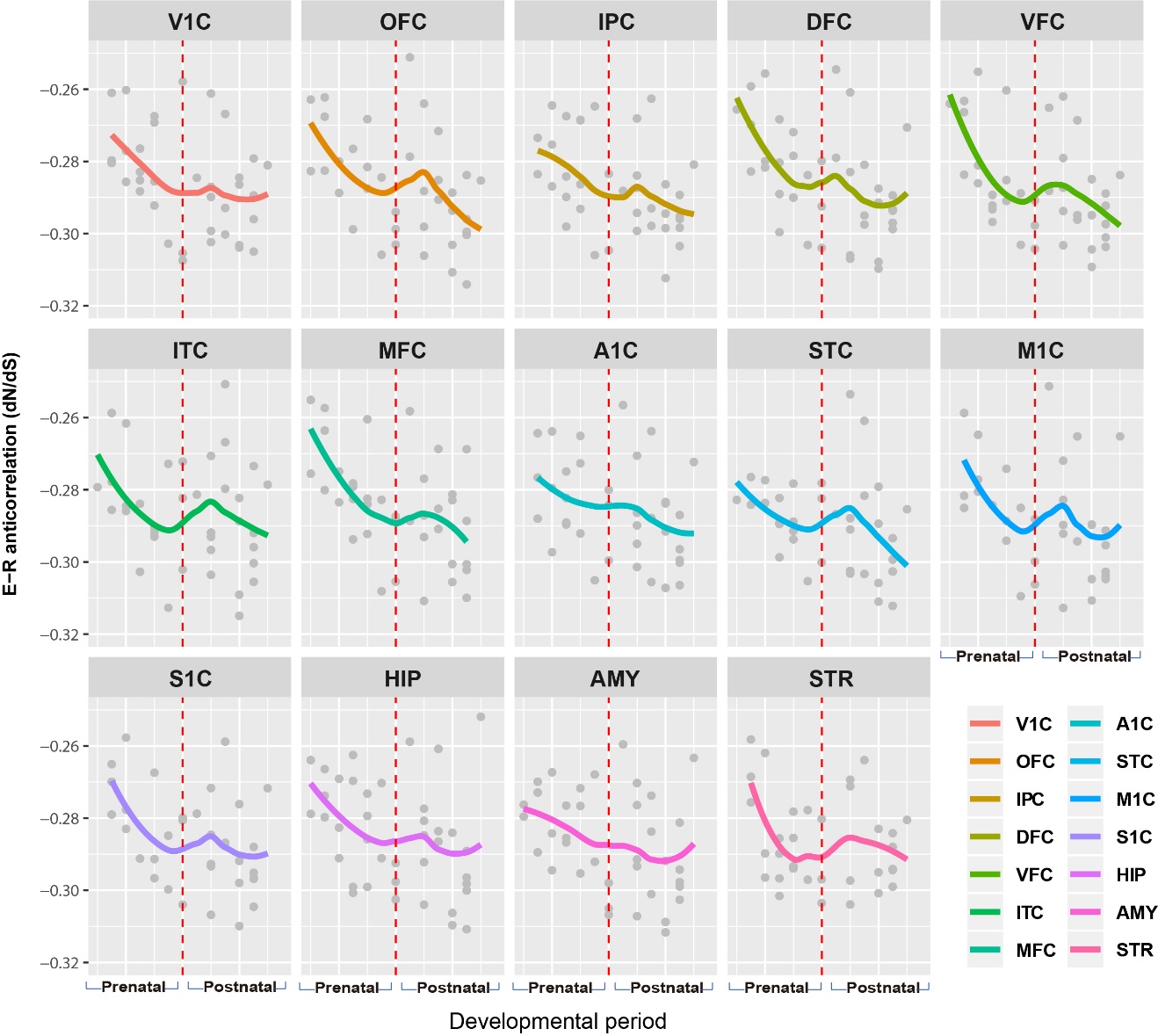


Fig S11. Dynamic changes of E-R anticorrelations during human brain development across different brain regions.

**Supplementary codes.** R codes used in the key analysis of the manuscript, including the calculation of E-R anticorrelation

########################## Tasic et al., 2016 data

setwd("~/Tasic et al")

dns=read.csv("Mouse_rat_dNdS22_aggregrated.csv",header=T)

dns=dns[dns[,2]!="Inf",]

type=read.csv("final_type.csv",header=T)

rownames(type)=type[,1]

type=type[,-1]

type[,9]=gsub("/","-",type[,9])

final_type=factor(type[,9])

exp=read.csv("1679 cells pass QC RPKM.csv",header=T)

rownames(exp)=exp[,1]

exp=exp[,-1]

exp2=exp[rownames(exp)%in%intersect(rownames(exp),dns[,1]),]

exp2[,1680]=dns[match(rownames(exp2),dns[,1]),3]

exp2[,1681]=dns[match(rownames(exp2),dns[,1]),4]

exp2[,1682]=dns[match(rownames(exp2),dns[,1]),2]

colnames(exp2)[1680:1682]=c("dN","dS","dN/dS")

cors_dn=function(x) cor(as.numeric(x),as.numeric(exp2[,l+1]),method="spearman")

pvalue_dn=function(x) cor.test(as.numeric(x),as.numeric(exp2[,l+1]),method="spearman")$p.value

cors_ds=function(x) cor(as.numeric(x),as.numeric(exp2[,l+2]),method="spearman")

pvalue_ds=function(x) cor.test(as.numeric(x),as.numeric(exp2[,l+2]),method="spearman")$p.value

cors_dns=function(x) cor(as.numeric(x),as.numeric(exp2[,l+3]),method="spearman")

pvalue_dns=function(x) cor.test(as.numeric(x),as.numeric(exp2[,l+3]),method="spearman")$p.value

coefficient=function(x)coefficients(lm(as.numeric(x)~as.numeric(exp2$`dN/dS`)))[2]

l=length(colnames(exp))

f=data.frame(matrix(nrow=l))

f[,1]=apply(exp2[,1:l],2,cors_dn)

f[,2]=apply(exp2[,1:l],2,pvalue_dn)

f[,3]=p.adjust(f[,2],method="fdr")

f[,4]=apply(exp2[,1:l],2,cors_ds)

f[,5]=apply(exp2[,1:l],2,pvalue_ds)

f[,6]=p.adjust(f[,5],method="fdr")

f[,7]=apply(exp2[,1:l],2,cors_dns)

f[,8]=apply(exp2[,1:l],2,pvalue_dns)

f[,9]=p.adjust(f[,8],method="fdr")

f=f[,-c(2,5,8)]

rownames(f)=colnames(exp)

rownames(anno)=gsub("X","",rownames(anno))

f[,7]=colSums(exp>0)

colnames(f)=c("dN","dn_p.adjust","dS","ds_p.adjust","dN/dS","dns_p.adjust","Gene_number")

f[,8]=residuals(lm(f$`dN/dS`~f$Gene_number))

colnames(f)[8]="corrected E-R anticorrelation_dn.ds"

f[,9]=residuals(lm(f$dN~f$Gene_number))

colnames(f)[9]="corrected E-R anticorrelation_dn"

f[,10:11]=type[match(rownames(f),rownames(type)),9:10]

colnames(f)[10:11]=c("subtype","class")

f[,12]=apply(exp2[,1:1679],2,mean)

colnames(f)[12]="mean"

f[,13]=residuals(lm(f$`dN/dS`~f$mean))

colnames(f)[13]="residual_dn.ds_mean"

f[,14]=apply(exp2[,1:1679],2,coefficient)

colnames(f)[14]="slope"

cor2_dns=f[!f$class%in%c("VLMC","Endo"),c(8,9,11)]

cor2_dns$class=factor(cor2_dns$class,levels=unique(cor2_dns$class)[c(2,1,5,3,4)])

cor2_dns=melt(cor2_dns,id="class")

library(ggplot2)

library(reshape2)

library(ggpubr)

library(Rmisc)

compare_means(value~class , data =cor2_dns)

postscript("~/Tasic 2016 E-R anticorrelation among different cell types-mean residual.eps",width=5,height=5)

ggboxplot(cor2_dns,x="class", y="value",fill="variable")+

ylab("corrected E-R anticorrelation")+

xlab("Cell type")+

stat_compare_means()+

theme( strip.text.x = element_text(size=14, face="bold"),

axis.text.x=element_text(hjust=1,size=10, face="bold"),

axis.title.y=element_text(size=12, face="bold"))

dev.off()

compare_means(slope~class , data =cor2_dns)

my_comparisons <- list( c("Glutamatergic", "GABAergic"),c("GABAergic", "Oligo"),c("Oligo","Astro"),c("Astro","Microglia"))

#####OMIM disease related genes E-R anticorrelations

disease=read.csv("~/OMIM_mental_disease_genes.csv",header=T)###428 genes

disease_genes=disease[,2]

cors_dn2=function(x) cor(as.numeric(x),as.numeric(exp3[,l+1]),method="spearman")

pvalue_dn2=function(x) cor.test(as.numeric(x),as.numeric(exp3[,l+1]),method="spearman")$p.value

cors_dns2=function(x) cor(as.numeric(x),as.numeric(exp3[,l+3]),method="spearman")

pvalue_dns2=function(x) cor.test(as.numeric(x),as.numeric(exp3[,l+3]),method="spearman")$p.value

l=length(colnames(exp))

f2=data.frame(matrix(nrow=l))

f2[,1]=apply(exp3[,1:l],2,cors_dn2)

f2[,2]=apply(exp3[,1:l],2,pvalue_dn2)

f2[,3]=p.adjust(f2[,2],method="fdr")

f2[,4]=apply(exp3[,1:l],2,cors_dns2)

f2[,5]=apply(exp3[,1:l],2,pvalue_dns2)

f2[,6]=p.adjust(f2[,5],method="fdr")

f2=f2[,-c(2,5)]

rownames(f2)=colnames(exp)

f2[,5]=type[match(rownames(f2),rownames(type)),10]

colnames(f2)=c("dN","dn_p.adjust","dN.dS","dns_p.adjust","Class")

f2=f2[f2$Class!="Endo",]

exp3=exp2[toupper(rownames(exp2))%in%disease_genes,] ###disease gene expression in diffent cells

exp3=as.data.frame(t(exp3))

exp3[,308]=type[match(rownames(exp3),rownames(type)),10]

colnames(exp3)[308]="type"

exp3=aggregate(exp3[,-308],by=list(exp3$type),FUN="mean")

exp4=melt(exp3,id="Group.1")

compare_means(value~Group.1 , data =exp4)

my_comparisons <- list( c("Glutamatergic", "GABAergic"),c("GABAergic", "Oligo"),c("Oligo","Astro"),c("Astro","Microglia"))

exp4$Group.1=factor(exp4$Group.1,levels=unique(exp4$Group.1)[c(4,3,6,1,5,2)])

ggboxplot(exp4,x="Group.1", y="value",fill="Group.1")+

ylab("mean RPKM")+

xlab("Cell type")+

stat_compare_means(comparisons = my_comparisons)+

stat_compare_means(label.y = -0.2)+

theme( strip.text.x = element_text(size=14, face="bold"),

axis.text.x=element_text(hjust=1,size=10, face="bold"),

axis.title.y=element_text(size=12, face="bold"))

f2$Class=factor(f2$Class,levels=unique(f2$Class)[c(2,1,5,3,4)])

compare_means(dN.dS~Class , data =f2)

my_comparisons <- list( c("Glutamatergic", "GABAergic"),c("GABAergic", "Oligo"),c("Oligo","Astro"),c("Astro","Microglia"))

postscript("~/Tasic 2016 diease genes E-R anticorrelation among different cell types.eps",width=5,height=5)

ggboxplot(f2,x="Class", y="dN.dS",fill="Class")+

ylab("E-R antocorrelation for disease genes")+

xlab("Cell type")+

stat_compare_means(comparisons = my_comparisons)+

stat_compare_means(label.y = -0.2)+

theme( strip.text.x = element_text(size=14, face="bold"),

axis.text.x=element_text(hjust=1,size=10, face="bold"),

axis.title.y=element_text(size=12, face="bold"))

dev.off()

################################################## mouse atlas data analysis

setwd("~/GSE108097_MCA_Figure2_BatchRemoved_dge.txt/")

exp=read.table("Figure2-batch-removed.txt",header=T)

exp3=exp[apply(exp,1,max)>0,] #eliminate rows gene expression all 0

tissue=gsub("_[0-9][.][A-Z]+","",colnames(exp3))

tissue=factor(tissue)

exp2=exp3[rownames(exp3)%in%intersect(dns[,1],rownames(exp3)),]

l=length(colnames(exp3))

exp2[,l+1]=dns[match(rownames(exp2),dns[,1]),3]

exp2[,l+2]=dns[match(rownames(exp2),dns[,1]),4]

exp2[,l+3]=dns[match(rownames(exp2),dns[,1]),2]

colnames(exp2)=c(colnames(exp3),"dn","ds","dn/ds")

cors_dns=function(x) cor(as.numeric(x),as.numeric(exp2[,l+3]),method="spearman")

pvalue_dns=function(x) cor.test(as.numeric(x),as.numeric(exp2[,l+3]),method="spearman")$p.value

f=data.frame(matrix(nrow=l))

f[,1]=apply(exp2[,1:l],2,cors_dns)

f[,2]=apply(exp2[,1:l],2,pvalue_dns)

f[,3]=p.adjust(f[,2],method="fdr")

f[,4]=as.character(tissue)

f[,5]=colSums(exp3>0)

colnames(f)=c("cor_dns","p_dns","p_dns_adj","tissue","gene_number")

rownames(f)=colnames(exp)

f[,6]=residuals(lm(f$cor_dns~f$gene_number))

colnames(f)[6]="corrected E-R anticorrelation"

write.csv(f,"microwell-seq-dns-cor-all-spearman 11-5-2019.csv")

setwd("~/GSE108097_MCA_Figure2_BatchRemoved_dge.txt/MCA")

library(Seurat)

#http://satijalab.org/seurat/mca.html Seurat Guided Clustering of the Microwell-seq Mouse Cell Atlas

mca.matrix <- readRDS(file = "./MCA_merged_mat.rds")

mca.metadata <- read.csv("./MCA_All-batch-removed-assignments.csv", row.names = 1)

mca <- CreateSeuratObject(counts = mca.matrix, meta.data = mca.metadata)

mca <- subset(mca, cells = rownames(mca@meta.data[!is.na(mca@meta.data$ClusterID),]), do.clean = TRUE)

#perform standard log-normalization.

mca <- NormalizeData(object = mca, normalization.method = "LogNormalize", scale.factor = 10000)

#FindVariableGenes

mca <- FindVariableFeatures(object =mca, selection.method = "vst", nfeatures = 1000, verbose = FALSE)

mito.genes <- grep(pattern = "^mt-", x = rownames(x = mca@assays$RNA), value = TRUE)

percent.mito <- Matrix::colSums(mca@assays$RNA[mito.genes, ])/Matrix::colSums(mca@assays$RNA)

mca <- AddMetaData(object = mca, metadata = percent.mito, col.name = "percent.mito")

mca <- ScaleData(object = mca, features = VariableFeatures(object = mca))

mca <- RunPCA(object = mca, pc.genes = hv.genes, pcs.compute = 100, do.print = TRUE, pcs.print = 1:5, genes.print = 5)

PCElbowPlot(object = mca, num.pc = 100)

mca <- FindClusters(object = mca, reduction.type = "pca", dims.use = 1:75, resolution = 3, save.SNN = TRUE, n.start = 10, nn.eps = 0.5, print.output = FALSE)

mca <- RunTSNE(object = mca, reduction.use = "pca", dims.use = 1:75, tsne.method = "Rtsne",

nthreads = 4, reduction.name = "tsne", reduction.key = "tSNE_",

max_iter = 2000)

tsne=as.data.frame(mca@reductions$tsne@cell.embeddings) ######save cell position coordinate on tsne plot

p[,3]=scale(f$"corrected E-R anticorrelation")

colnames(p)[3]="corrected E-R anticorrelation "

library(shape)

library(gplots)

breaks=quantile(unique(p$"corrected E-R anticorrelation"),probs = c(seq(0,0.2,by=0.01),seq(0.21,1,by=0.02)))

labels=1:60

mapcolor=colorpanel(60,low="red",mid="yellow",high="gray")

exp=cut(unique(p$"corrected E-R anticorrelation"),breaks,labels=labels,include.lowest=T,right=F)

########## map corrected E-R anticorrelation back to tsne

png("Fig 2A tsne plot with corrected E-R anticorrelationas colors.png",width=8,height=20,units="in",res=350)

plot(x=p[,1],y=p[,2],col=mapcolor[exp],pch=46,main="corrected E-R anticorrelation (dN/dS)",xlab="tSNE-1",ylab="tSNE-2")

col.labels=round(c(min(breaks),mean(breaks),max(breaks)),2)

colorlegend(col=mapcolor,zlim=round(c(min(breaks),max(breaks)),2),zlevels=50,zval=col.labels,posx=c(0.92,0.94),main.cex=0.7,digit=1,left=FALSE)

dev.off()
